# Age-Period-Cohort Analysis on the Burden of Gastrointestinal Cancers in China: Trends, Risk Factors, and Predictions

**DOI:** 10.3390/healthcare13101096

**Published:** 2025-05-08

**Authors:** Yongbo Lu, Jingya Zhang, Zongyang Zhou, Ning Zhang, Wei Ning, Ying Mao

**Affiliations:** 1 School of Public Policy and Administration, Xi’an Jiaotong University, Xi’an 710049, China; 2 Vanke School of Public Health, Tsinghua University, Beijing 100084, China

**Keywords:** gastrointestinal cancer, incidence, risk factors, age–period–cohort model, prediction

## Abstract

Background: Gastrointestinal cancer imposes a heavy public health burden worldwide. The six major subtypes of GI cancer cases accounted for over a quarter of the total cancer cases in China. We examined and predicted the amount and trend of the burden of gastrointestinal cancer in China. Methods: We collected the crude incidence rate (CIR), age-standardized incidence rate (ASIR), age-standardized prevalence rate (ASPR), crude DALY rate, age-standardized DALY rate, crude death rate (CDR), and age-standardized death rate (ASDR) by gender, age, and risk factors from the Global Burden of Disease 2021 database, covering the period from 1990 to 2021. We used an age–period–cohort (APC) model to calculate the age, period, and cohort effects separately and a Bayesian APC model to predict the epidemiological trends and the age-specific incidence. Results: In 2021, 1,957,948 incidence cases of gastrointestinal cancers were estimated in China, with an incidence rate of 137.62 per 100,000. The burden was notably higher for males, with tobacco use accounting for 26.48% of male GI cancer DALYs versus dietary risks (13.22%) being predominant in females. Metabolic risk factors showed concerning growth trends, with high fasting plasma glucose increasing by 166.06% in males and 275.72% in females. The age–period–cohort analysis revealed peak incidence at advanced ages (>80 years) for most GI cancers. By 2050, China’s gastrointestinal cancer burden is projected to increase substantially, with total cases reaching 2.92 (95% CI: 2.27, 3.77) million (49.06% increase) and crude incidence rate rising to 230.51 (95% CI: 178.98, 298.03) per 100,000 (a 67.49% increase), predominantly driven by colorectal cancer (41.74%) and stomach cancer (21.47%). While crude incidence rates show consistent increases across all GI cancers, particularly in males, age-standardized incidence rates are predicted to decrease for most cancer types except for male colorectal and biliary tract cancers. Conclusions: The burden of gastrointestinal cancer in China is growing rapidly and will continue to increase. The performance of ASIR indicated that population aging is a potential driver of the increasing burden. Cancer control policies should focus on older people. It is critical to distinguish prevention strategies by gender and age according to the major risk factors.

## 1. Introduction

Gastrointestinal (GI) cancers form in the lining of the gastrointestinal tract, such as the esophagus, liver, stomach, pancreas, intestine, and rectum, imposing a heavy public health burden worldwide. Generally speaking, GI cancers created a bigger burden among males and the elderly. The prognosis of most GI cancers, except for colorectal cancer, was poor due to late diagnosis. Established risk factors of GI cancers include tobacco and alcohol use, diet, obesity, physical activity, and viral infections. The development of all GI cancers is highly associated with lifestyle choices, and all can be detected by identifying precancerous diseases [1].

According to the GLOBOCAN 2022 [2] database, there were 5 million estimated newly diagnosed GI cancer cases in 2022, accounting for approximately one-fourth of the cancer cases. Globally, the estimated age-standardized incidence of colorectal cancer ranked highest among GI cancers, with the age-standardized incidence being 17.8 per 100,000, followed by stomach cancer (9.2/100,000, 968,350), liver cancer (8.6/100,000, 865,269), esophagus cancer (5.0/100,000, 510,716), pancreas cancer (4.7/100,000, 510,566), and gallbladder cancer (1.2/100,000, 122,462); other types are much less common, including neuroendocrine tumors, gastrointestinal stromal tumors, and anal cancer. The total incidence of GI cancers is higher than most common lung cancers and is expected to grow rapidly, especially in developing countries. From 2022 to 2045, the new cases and deaths are estimated to increase by 66.4% and 75.5%, respectively, due to ongoing population aging and growth.

The burden of gastrointestinal cancers differs greatly geographically, which has been acknowledged by many existing studies [3,4]. Esophageal, gastric, and liver cancers were more common in Asia than in other parts of the world [5,6,7]. The incidence of colorectal and pancreatic cancers was highest in Europe and North America [8,9], while the burden of gallbladder cancer was higher in South America [10]. In China, it was estimated that these six major subtypes of GI cancer cases reached 1,617,251 cases in 2022, accounting for roughly 33.5% of the total cancer cases. Many studies have focused on the epidemiology and risk factors of GI cancers in China. For instance, Liu et al. reviewed the incidence and mortality of colorectal cancer in China in 2011 based on cancer registry data. Cui et al. investigated the temporal and spatial patterns of the epidemiological characteristics of esophageal cancer in Guangzhou during 2012–2017 and explored the association between the epidemiological characteristics of esophageal cancer and potential influencing factors. Lin et al. reviewed the epidemiology of and urban/rural disparities in pancreatic cancer burden in China. Zheng et al. identified cigarette smoking, family history, obesity, and diabetes to be risk factors for pancreatic cancer. In China, the major risk factors for gastrointestinal cancers include a high consumption of salted, pickled, and smoked foods, as well as tobacco smoking and heavy alcohol use [11,12]. In contrast, in Western countries, rising obesity rates and the increasing prevalence of gastroesophageal reflux disease are the predominant contributors to gastric and esophageal adenocarcinomas. These regional differences in risk factor profiles highlight the need for tailored prevention strategies across populations.

With the increasing burden of GI cancers, there was tremendous interest in understanding the disease burden of different types of GI cancers in China [13,14], but few studies have focused on the comparison and prediction of the burden of GI cancers in China. To fill this research void, the present study aims to review, forecast, and compare the epidemiologic burden of six major types of GI cancers in China, namely cancers of the esophagus, stomach, colorectum, liver, pancreas, and gallbladder. Due to the huge differences in male and female GI cancer burden in China, we further compared the GI cancers by gender and age groups. By carrying this out, this study adds value to the existing literature and provides additional evidence to help health leaders and policymakers improve their understanding of GI cancer epidemiology, identify populations at high risk, and develop prevention and control strategies.

## 2. Materials and Methods

### 2.1. Data Source

Gastrointestinal cancer (including stomach cancer, esophageal cancer, gallbladder and biliary tract cancer, pancreatic cancer, liver cancer, and colon and rectum cancer) burden and population data were obtained from the “Global Burden of Disease Study 2021” (GBD 2021) at http://ghdx.healthdata.org/gbd-results-tool (accessed on 16 May 2024). The GBD 2021 database was launched and developed by the Institute for Health Metrics and Evaluation (IHME). All the data were calculated through estimation based on a systematic review. The estimation process and method were published online (http://ghdx.healthdata.org/gbd-2021/code/nonfatal-2 (accessed on 16 May 2024)). We searched and collected the crude incidence rate (CIR), age-standardized incidence rate (ASIR), crude prevalence rate (CPR), age-standardized prevalence rate (ASPR), crude DALY rate, age-standardized DALY rate, crude death rate (CDR), and age-standardized death rate (ASDR) by gender, age, and risk factors from 1990 to 2021. Population data were collected as estimates for 5-year-interval age groups in China for the study period.

Although the Global Burden of Disease (GBD) 2021 data exhibit inherent limitations regarding variable data quality across regions, heightened uncertainty in data-sparse contexts, and incomplete quantification of mediating pathways between risk factors that may potentially impact the precision of predictive analyses [15], these data nonetheless remain the most comprehensive, methodologically rigorous, and internationally recognized resource for global disease burden assessment, providing essential epidemiological evidence for health policy formulation across multiple governance levels [16].

### 2.2. Statistical Analysis

We employed the age–period–cohort (APC) model to analyze disease incidence patterns using the natural logarithm of incidence as the dependent variable and the median of datasets as the independent variable. This approach allowed us to isolate three distinct temporal effects: (a) Age effect: This represents physiological and pathological changes associated with aging that influence disease incidence. The longitudinal age coefficient depicts fitted age-specific rates relative to reference cohorts and is adjusted for period deviations. (b) Period effect: This captures temporal changes in disease incidence caused by various events. Period rate ratios (RRs) compare age-specific rates in a given period to those in the reference period. (c) Cohort effect: This reflects generational differences in disease mortality rates resulting from lifestyle changes or varying exposure levels to risk factors (e.g., dietary structures, cooking habits, and living environments). Cohort RRs compare age-specific rates between cohorts. Additionally, we calculated net drift to estimate the annual percentage change in expected age-adjusted rates over time.

To forecast gastrointestinal cancer trends, we implemented a Bayesian APC model, which assumes similarity between close time effects through prior probability distributions. This hierarchical approach incorporates uncertainty about hyperparameters and addresses identifiability problems by applying mildly informative prior distributions. Using these models, we calculated age-specific incidence and applied the 2000 GBD population demographic structure for standardization to project age-standardized incidence rates (ASIR) for 2022–2050.

### 2.3. Software

The APC analysis was completed using the web tool from the National Cancer Institute (https://analysistools.cancer.gov/apc/ (accessed on 21 Aug 2014)). The bamp package of R (version 4.4.2) was used to predict the incidence of gastrointestinal disease. All the figures were created using the ggplot2 package in R (version 4.4.2). The selection of analytical tools was based on their methodological robustness and widespread validation in epidemiological research [17,18,19].

## 3. Results

### 3.1. The Burden of Gastrointestinal Cancers in China

In 2021, 1,957,948 new cases of gastrointestinal cancer were estimated, with an incidence rate of 137.62 per 100,000 in GBD 2021. In the context of gender, the incidence of gastrointestinal cancers was much higher among males than females. In detail, for specific cancers, a steady upward trend was noted for the incidence of stomach cancer, gallbladder and biliary tract cancer, pancreatic cancer, and colon and rectum cancer between 1990 and 2021. The incidence of these four cancers increased by 132.06% over a 32-year period. In detail, colon and rectum cancers are the most common gastrointestinal cancer type, with 658.32 thousand cases and 46.27 cases per 100,000 in 2021. It is worth noting that the incidence of colon and rectum cancer in China has risen dramatically by 473.92% in this period. Apart from that, the incidence of liver cancer exhibited a marked decline between 2000 and 2005, followed by a gradual rise. This was subsequently followed by a decline, albeit a gradual one, between 2019 and 2021. The absolute value and percentage change in ASIR were significantly lower than CIR (Table 1).

From the perspective of gender difference, the burden of the disease for males is generally higher than that of females. In 2021, the incidence of six kinds of gastrointestinal cancers in men was 2.16 times higher than that in women. The incidence of esophageal, stomach, liver, pancreatic, and colon and rectum cancer has been significantly higher in males than in females. In 1990, the incidence of gallbladder and biliary tract cancer in females was higher than that in males, but it has changed since 2009. For all cancers, the incidence growth rate was higher in men than in women between 1990 and 2021 (Table 1).

### 3.2. Attributable Risk Factors for Gastrointestinal Cancers

The GBD estimated major measurable risk factors for the disease. Risk factors for gastrointestinal cancers mainly include behavioral factors (tobacco, high alcohol use, dietary risks, drug use, and low physical activity) and metabolic factors (high fasting plasma glucose and high body mass index).

**Figure 1:**
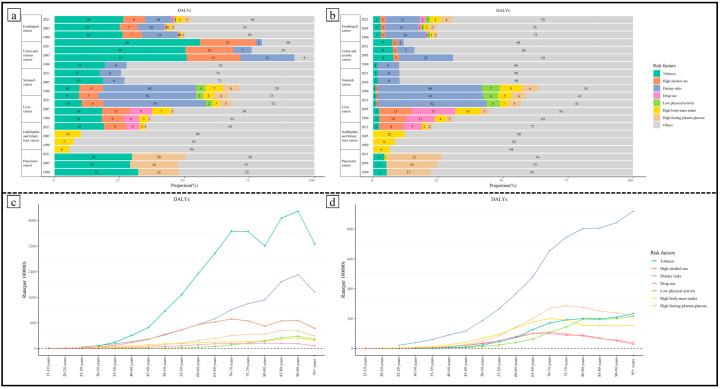
(**a**) Age-standardized DALY rate attributed to various risk factors in 1990, 2005, and 2021 for males. (**b**) Age-standardized DALY rate attributed to various risk factors in 1990, 2005, and 2021 for females. (**c**) Age-standardized DALY rate attributed to various risk factors by age in 2021 for males. (**d**) Age-standardized DALY rate attributed to various risk factors by age in 2021 for females.

In 2021, the leading risk factor in males in terms of attributable cancer DALYs was tobacco (Figure 1), which accounted for 26.48% of all male gastrointestinal cancer DALYs. The DALY rate attributed to tobacco increased by 17.18% from 1990 to 2021. Dietary risk and high alcohol use were the next greatest factors, accounting for 10.01% and 8.37% of all male gastrointestinal cancer DALY rates in 2021, respectively, followed by high FPG (2.97%, which increased by 166.06%). For females, dietary risk was the leading risk factor for gastrointestinal cancer DALYs in China as well as globally in 2021, accounting for 13.22% of all female gastrointestinal cancer DALY rates. And the female gastrointestinal cancer DALY rate attributed to dietary risk had decreased by 18.90% from 1990 to 2021. High FPG and high BMI were the next risk factors and accounted for 4.45% and 4.11% of all female gastrointestinal cancer DALY rates (with increases of 275.72% and 313.22%, respectively, from 1990 to 2021), followed by tobacco, which accounted for 2.60% of that. The DALY rate attributed to behavioral factors decreases when we use an age-standardized index, while DALYs attributed to metabolic factors still increase. The ranking and trends of these risk factors by attributable gastrointestinal cancer deaths (and age-standardized deaths) in 2021 showed a similar ranking to DALYs (and age-standardized DALYs) (Figure 1a,b).

Age-specific analysis revealed age-related heterogeneity in the importance of various risk factors for gastrointestinal cancers. For males, tobacco, high alcohol use, and dietary risks were the leading risk factors across all age groups. In the under-65 age groups, high alcohol use and dietary risks contributed similarly to gastrointestinal cancer DALYs; in the 65+ age groups, the importance of dietary risks significantly surpassed that of high alcohol use. The contribution of high fasting plasma glucose (FPG) gradually increased with age, exceeding BMI to become the fourth leading risk factor for gastrointestinal cancers in the 55+ age groups. Another noteworthy point is that the contribution of low physical activity to gastrointestinal cancer DALYs rapidly rose in the elderly group, making it the fifth most significant risk factor for gastrointestinal cancer DALYs in those over 80 years old. For females, dietary risks were the primary risk factor in age groups above 25 years old. The contribution of high FPG surpassed that of high BMI to become the second leading risk factor for gastrointestinal cancers in the 60–64 age group. In contrast to males, the contribution of tobacco increased rapidly only in age groups over 60, becoming the third leading risk factor for the 70 to 94 age group. Physical inactivity was more important than high BMI in the 80+ age group. The contribution of high alcohol use and drug use continuously declined above the age of 65 (Figure 1c,d).

### 3.3. APC Model Analysis of Gastrointestinal Cancer Incidence in China

We fit an APC model to examine trends in overall gastrointestinal and liver cancer incidence rates (Table 2). For the same period and cohort, incidence rates of colon and rectum cancer, gallbladder and biliary tract cancer, pancreatic cancer, and stomach cancer showed a steep increase by age among males and females, and the incidence rate among males was higher overall than that among females. The age groups with the highest incidence rate were mainly those aged 80 years. For males, specifically, it reached its highest incidence at 90–94 for all six kinds of gastrointestinal cancers. For females, it reached its highest incidence at 90–94 for pancreatic cancer and 85–89 for other gastrointestinal cancers. The incidence of liver cancer first decreased and then increased with age. In young age groups, the incidence rates in men were lower than those in women for all cancers, but this is in contrast to upper age groups.

Then, we took the period 2002–2006 as a baseline to analyze the trend by years. The period effects were estimated as non-steady linear trends. Overall, the incidence rate of colon and rectum cancer, gallbladder and biliary tract cancer, and pancreatic cancer for both genders increased over the years; meanwhile, that of esophageal cancer and stomach cancer decreased. For females, the incidence rate of liver cancer decreased, and that for males tended to fluctuate during 1992–2021.

In terms of cohort, we selected cohort 1952 as the baseline to estimate the rate ratio. The cohort effects were confirmed as linear trends. The incidence rate of colon and rectum cancer, gallbladder and biliary tract cancer, and pancreatic cancer showed a steady increase by cohort for males, while that of esophageal cancer and stomach cancer decreased. The incidence rate of esophageal cancer, stomach cancer, and colon and rectum cancer by cohort for females appeared similar to males. Different from the trend in male groups, the incidence of gallbladder and bile duct cancer and pancreatic cancer in women rose first and then declined by cohort. The incidence rate of liver cancer for both genders first increased and then decreased by cohort in 1992–2021.

### 3.4. Prediction Based on the Bayesian Age–Period–Cohort Model

We made predictions of crude incidence rates until 2050 and used GBD population projections to estimate the incidence case number. The incidence of gastrointestinal cancer was predicted to increase to 2,918,453 [2,266,081, 3,773,383] by 49.06% in 2050; meanwhile, the crude incidence rate is predicted to rise to 230.51 [178.98, 298.03] per 100,000 by 67.49%. The incidence of colon and rectum cancer and stomach cancer mainly accounts for 41.74% and 21.47% of total cases. Specially, the highest crude incidence cancer rate is colon and rectum cancer at 96.21 [74.49, 123.47] per 100,000, followed by stomach cancer (63.31 [50.24, 80.33] per 100,000), esophageal cancer (26.99 [19.81, 37.79] per 100,000), liver cancer (21.47 [15.68, 29.54] per 100,000), pancreatic cancer (14.96 [12.56, 17.75] per 100,000), and gallbladder and biliary tract cancer (7.56 [6.20, 7.56] per 100,000).

**Figure 2:**
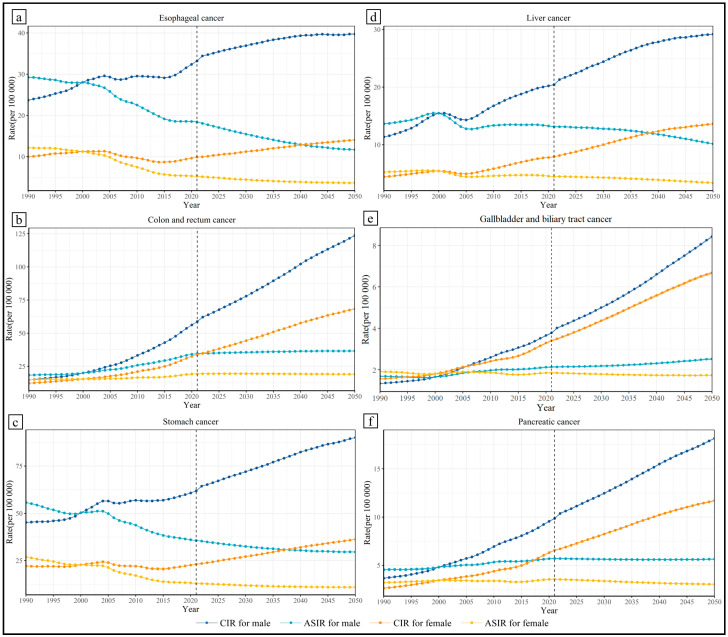
Predicted crude incidence rate and age-standardized incidence rate.

From the perspective of gender differences, the burden of gastrointestinal cancers for the male group is still much higher than the female group in 2050 (Figure 2). A steady upward trend of CIR existed in gastrointestinal cancers for all genders from 2021 to 2050. The CIRs of colon and rectum cancer, as well as gallbladder and biliary tract cancer, for males in 2050 will be more than two times those in 2021. Then, the results were standardized with population data from 2000 in China. The ASIR of all six kinds of gastrointestinal cancers for females is predicted to decrease from 2021 to 2050. For males, the ASIR of colon and rectum cancer and gallbladder and biliary tract cancers still shows an apparent increase in the prediction period, while that of the other gastrointestinal cancers decreased.

Furthermore, the age and predicted CIR are basically positively correlated (Supplementary Figure S1a–l). In 2050, for males, the age group with the highest CIR of six kinds of gastrointestinal cancers is 90–94 years. In detail, the highest CIR (per 100,000) in 2050 and the rate of increase during the prediction duration are as follows: esophageal cancer, 305.89 [231,23, 414.29], −4.50%; stomach cancer, 490.01 [396.68, 612.19], −9.50%; colon and rectum cancer, 736.54 [553.50, 966.30], 56.27%; liver cancer, 132.49 [97.52, 180.60], 13.48%; gallbladder and biliary tract cancer, 52.52 [42.06, 64.79], −1.33%; and pancreatic cancer, 107.14 [90.16, 127.13], 9.88%. For females, the age group with the highest CIR (per 100,000) of esophageal cancer and stomach cancer is 95 plus years at 60.19 [40.42, 91.67] and 161.04 [124.17, 211.70], which decreased by 24.50% and 15.49%; the age group with the highest CIR (per 100,000) of pancreatic cancer is 90–94 years at 48.72 [41.12, 58.04], which decreased by 0.87%; the age group with the highest CIR (per 100,000) of colon and rectum cancer, liver cancer, and gallbladder and biliary tract cancer is 85–89 years at 243.14 [199.61, 296.60], 65.25 [47.52, 90.96], and 23.96 [20.23, 28.34], which increased by 20.53%, 19.35%, and −10.40%.

Apart from that, for males, the median age of incidence of colon and rectum cancer, as well as esophageal cancer, is 75–79 years, and that of stomach cancer, liver cancer, gallbladder and biliary tract cancer, and pancreatic cancer is 70–74 years. For females, the median age of incidence of all six kinds of gastrointestinal cancers is 75–79 years.

## 4. Discussion

To the best of our knowledge, this study is the first to systematically estimate the burden of GI cancers in China using CIR, ASIR, ASPR, ASDR, and DALYs. The findings indicated that gastrointestinal cancers were a significant health concern in China, with colon and rectum cancer being the most prevalent in 2021, followed by stomach, esophageal, liver, pancreatic, and gallbladder and biliary tract cancers. The absolute burden of GI cancers, measured by CIR, was found to increase significantly between 1990 and 2021, but age-standardized indices demonstrated a concurrent decrease. These divergent trends suggested that the rise in GI cancer burden might be driven by the aging population and shifts in the disease spectrum within China [20,21]. On the one hand, the overall burden of colon and rectum cancer, as well as pancreatic cancer, has been on the rise. Of these, the increasing prevalence of pancreatic cancer in China could be attributed to factors such as the growing trend in chronic diseases like diabetes, indicating a shift from the previous cholestatic pancreatic disorders [22,23]. In addition, alterations in dietary habits and a rise in sedentary lifestyles over the past several decades among the Chinese population have been proven to be correlated with an elevated prevalence of colon and rectum cancer [24,25]. On the other hand, esophageal cancer, liver cancer, and stomach cancer have demonstrated a notable declining trend that can be ascribed to the enhanced understanding and management of infections associated with these malignancies [26,27,28]. For instance, vaccinations against hepatitis B and advancements in hepatitis C treatment have contributed to a reduction in liver cancer incidence [26,29]. Similarly, the widespread availability and utilization of endoscopic screening have also facilitated the decline of both esophageal and stomach cancers [30,31], and the effective management of Helicobacter pylori infections has played a role in the decline of stomach cancer cases [28]. Notably, gallbladder and biliary tract cancer trends have remained stable. However, these trends and factors vary between sexes and age groups.

Our study also revealed a gender disparity in the distribution of gastrointestinal cancers, with men being more vulnerable to the disease than women. This finding is in line with the global trend and may reflect differences in lifestyle habits and hormone levels [32,33]. This means that, for men, there is more exposure to behavioral risk factors such as tobacco and alcohol, which are leading risk factors for GI burdens. The predominant risk factor in males was tobacco use, whereas dietary risk emerged as the most prominent risk factor for females. This indicates differences in patterns of disease between men and women. This disparity underlines the necessity for tailored prevention strategies that consider gender-specific risk factors to enhance their effectiveness [34,35]. In addition, sex steroid hormones, such as estrogen and progesterone, may play a protective role in reducing cancer risk in women [36,37]. The results of the APC analysis showed that the period effects of esophageal cancer in women decreased, which was the opposite of that in men. This suggests that the gap in esophageal cancer burden is widening, which is due to esophageal cancer being more likely to be inherited from same-sex relatives [38]. The results are instructive for clinic screening strategies.

The age-related trends in crude incidence rates (CIRs) reveal a complex interplay between age and cancer risk. While the general trend shows a positive correlation between age and CIR, there are some variations depending on the specific cancer type. This complexity underscores the need for age-specific strategies that consider the unique risks and needs of different age groups [33,39]. For example, screening programs should be tailored to target high-risk age groups for each cancer type, and healthcare professionals should be trained to recognize and address the unique challenges faced by older adults with cancer, such as comorbidities, polypharmacy, and the need for specialized care [40]. Furthermore, this study’s findings indicate a shift in the median age of incidence for various gastrointestinal cancers. This change in age distribution guides healthcare planning and resource allocation to note newer trends in these diseases [41,42]. As the population continues to age, the demand for cancer care services is likely to increase, placing additional strain on healthcare systems [41]. Policymakers must consider these demographic shifts when planning for future healthcare needs and invest in the necessary infrastructure, workforce, and resources to ensure adequate care for the growing number of older adults with cancer. Finally, the study’s projections for the future burden of gastrointestinal cancers emphasize the importance of ongoing monitoring and surveillance efforts [7,43]. Regularly tracking trends in cancer incidence and mortality can help identify emerging patterns and inform the development of targeted interventions [44].

Moreover, continued research into the causes, risk factors, and potential prevention strategies for gastrointestinal cancers is essential to reducing their impact on public health. Smoking, alcohol consumption, poor diet, obesity, and infections were identified as potential risk factors contributing to the high incidence and mortality rates of gastrointestinal cancers. Moreover, the increase in DALYs attributed to metabolic risk factors, such as high BMI, across age-standardized indices is particularly concerning. The study’s findings suggest that metabolic risk factors have a growing impact on GI cancer burden, indicating that future public health efforts should place a higher emphasis on addressing modifiable metabolic risk factors [45]. These efforts may include promoting healthy dietary practices [46], encouraging regular physical activity [35,47], and supporting weight management programs [39]. The decrease in DALYs attributed to behavioral factors when using an age-standardized index also warrants attention. Although behavioral factors still contribute significantly to the burden of GI cancers, this trend may indicate that public health interventions targeting behavioral factors, such as anti-smoking campaigns, have had some success in mitigating their impact [14,48]. Nevertheless, it is crucial to continue monitoring these trends and refining interventions to ensure sustained progress in reducing the burden of GI cancers.

The age-related variation in the importance of risk factors is a critical finding with potential implications for the design and implementation of targeted public health interventions. In particular, the notable differences in the ranking of risk factors between younger and older age groups warrant further investigation. The paper highlights the significance of dietary risk, alcohol use, and high body mass index as the top three attributable factors for all genders in the younger age group. However, in older age groups, tobacco use emerges as a dominant risk factor, especially for males over 65 years of age, which may be related to their mood changes after retiring [49]. This finding suggests that age-specific prevention strategies may be essential in mitigating the disease burden in different age groups.

The findings highlighted the projected increase in the burden of gastrointestinal cancers until 2050, with a 175.36% increase in cases and a 194.17% increase in crude incidence rates. One key observation in the study is the growing importance of colon and rectum cancer as well as stomach cancer, which are predicted to account for 49.49% and 26.53% of total cases, respectively. The high crude incidence rates for these cancers, particularly colon and rectum cancer, suggest the need for targeted interventions to reduce their impact [50,51]. These interventions may include promoting screening programs, improving access to healthcare, and encouraging lifestyle changes that can reduce the risk of developing these cancers [14,52]. For example, the Screening, Early Diagnosis, and Early Treatment program, initially introduced in rural regions of China in 2005 and later expanded to urban areas in 2013, is expected to have a significant effect on the incidence of colorectal cancer [14]. The program was also in line with the Asia Pacific Consensus Recommendations on colorectal cancer screening [53]. In terms of gender differences, the study predicts a persistently higher burden of gastrointestinal cancers for males compared to females in 2049. This disparity highlights the importance of understanding the biological, social, and behavioral factors contributing to these gender-specific trends [33,47]. For example, it is possible that specific genetic or hormonal factors make males more susceptible to certain gastrointestinal cancers [34]. Additionally, societal norms and expectations may influence health behaviors differently for everyone [43,54]. Addressing these questions will help inform the development of gender-specific prevention and control strategies. At the same time, we acknowledge that limitations of data quality and variable relationships may lead to potential bias risks in the predictions, as mentioned in the Methods Section.

This study acknowledged several limitations. First, our analysis did not include data on additional potential risk factors, such as genetics, occupation, and environmental pollution, which may substantially impact the burden of gastrointestinal cancers in China and necessitate further research. Second, while we assessed the overall disease burden of GI cancers in China, the absence of provincial data precluded the evaluation of regional differences in disease burden throughout the country.

## 5. Conclusions

Our study underscores the considerable burden of gastrointestinal cancers in China and the necessity of implementing effective measures to decrease both incidence and mortality rates associated with the disease. Our findings emphasize the importance of implementing early detection and screening programs, fostering healthy lifestyle habits, and mitigating the impact of urbanization on the disease burden. Additionally, reducing tobacco and alcohol consumption through efficacious policies holds significant potential in alleviating the burden of gastrointestinal cancers in China. Consequently, we strongly recommend that policymakers and health professionals focus on implementing interventions targeting the identified risk factors of the disease to achieve broader public health objectives, including reducing mortality rates and enhancing the quality of life for Chinese citizens.

## Figures and Tables

**Table 1:** The burden of gastrointestinal cancers in 2021 and its percentage change from 1990 to 2021 in China.

Cancer	Sex	CIR (95% CI)		ASIR (95% CI)		ASPR (95% CI)		ASDR (95% CI)		Age-Standardized DALY Rate (95% CI)	
		Value (per 100,000)	Percentage Change	Value (per 100,000)	Percentage Change	Value (per 100,000)	Percentage Change	Value (per 100,000)	Percentage Change	Value (per 100,000)	Percentage Change
Esophageal cancer	Both	22.55	27.84	15.04	−39.35	24.99	−28.32	14.13	−45.78	317.18	−51.45
		(18.00 to 27.75)	(−3.12 to 66.63)	(12.04 to 18.43)	(−53.47 to −21.89)	(19.95 to 30.69)	(−45.47 to −7.15)	(11.36 to 17.18)	(−58.18 to −30.43)	(252.46 to 392.42)	(−63.81 to −36.54)
	Female	10.24	−2.65	6.40	−55.70	12.15	−39.37	5.79	−62.02	114.58	−67.15
		(6.03 to 13.52)	(−29.39 to 38.54)	(3.76 to 8.47)	(−67.60 to −37.59)	(7.11 to 16.03)	(−56.89 to −12.22)	(3.39 to 7.58)	(−72.13 to −46.94)	(70.78 to 150.41)	(−76.49 to −51.80)
	Male	34.29	41.01	24.78	−31.29	38.59	−22.60	23.84	−37.58	534.18	−44.52
		(26.37 to 43.39)	(−0.24 to 93.49)	(19.19 to 31.20)	(−50.54 to −6.52)	(29.61 to 48.89)	(−45.00 to 6.57)	(18.51 to 29.79)	(−54.95 to −16.06)	(407.77 to 678.22)	(−60.66 to −23.78)
Stomach cancer	Both	43.00	24.15	29.05	−39.51	57.22	−14.81	21.51	−53.29	501.26	−57.58
		(33.17 to 53.81)	(−1.80 to 62.46)	(22.42 to 36.20)	(−52.04 to −21.45)	(44.18 to 71.99)	(−33.09 to 11.93)	(16.66 to 26.61)	(−62.74 to −39.75)	(387.29 to 627.98)	(−66.38 to −44.53)
	Female	23.81	5.22	15.23	−49.60	26.71	−33.77	12.02	−59.66	268.83	−63.82
		(18.39 to 29.96)	(−19.78 to 43.23)	(11.77 to 19.16)	(−61.35 to −31.55)	(20.68 to 33.89)	(−49.53 to −8.50)	(9.29 to 15.10)	(−68.89 to −45.71)	(208.91 to 340.98)	(−72.51 to −50.21)
	Male	61.31	33.56	44.48	−34.24	89.25	−5.82	32.61	−49.57	750.39	−54.10
		(44.76 to 80.93)	(−4.04 to 85.30)	(32.18 to 58.38)	(−51.87 to −9.96)	(65.15 to 117.49)	(−31.99 to 29.90)	(23.61 to 42.80)	(−62.98 to −31.08)	(550.90 to 997.91)	(−67.18 to −36.46)
Colon and rectum cancer	Both	46.27	243.69	31.44	65.13	168.62	141.23	13.64	−11.98	331.73	−15.08
		(37.39 to 56.09)	(158.33 to 344.63)	(25.53 to 37.97)	(24.50 to 111.85)	(136.58 to 203.05)	(86.27 to 208.99)	(11.09 to 16.31)	(−32.72 to 11.40)	(267.78 to 400.70)	(−36.19 to 9.58)
	Female	34.45	180.26	21.87	33.08	120.03	96.29	9.34	−29.43	220.00	−34.17
		(26.16 to 44.03)	(98.09 to 298.18)	(16.58 to 27.92)	(−5.56 to 88.33)	(92.27 to 153.21)	(40.04 to 174.56)	(7.10 to 11.88)	(−49.47 to −0.94)	(167.51 to 282.02)	(−53.68 to −5.59)
	Male	57.55	295.19	42.24	89.31	219.30	175.78	18.95	2.11	452.83	−0.28
		(43.93 to 74.39)	(173.94 to 465.16)	(32.56 to 54.26)	(31.97 to 166.55)	(169.59 to 280.99)	(95.59 to 287.01)	(14.65 to 24.34)	(−28.40 to 40.90)	(349.19 to 585.28)	(−30.81 to 42.22)
Liver cancer	Both	13.82	68.61	9.52	−10.04	13.29	−1.68	8.35	−22.35	239.91	−28.28
		(17.12 to 11.12)	(26.00 to 121.80)	(7.72 to 11.78)	(−32.28 to 17.51)	(10.75 to 16.41)	(−25.47 to 28.52)	(6.80 to 10.29)	(−41.21 to 0.71)	(191.98 to 299.37)	(−46.40 to −5.19)
	Female	7.61	65.25	4.89	−18.96	6.64	−15.59	4.57	−27.85	111.91	−36.94
		(5.91 to 9.65)	(21.76 to 130.45)	(3.82 to 6.18)	(−40.05 to 13.03)	(5.19 to 8.32)	(−37.26 to 16.32)	(3.57 to 5.76)	(−46.53 to −0.13)	(87.16 to 141.96)	(−53.79 to −11.66)
	Male	19.75	70.69	14.34	−4.83	20.00	5.61	12.40	−18.35	368.19	−23.92
		(14.96 to 26.62)	(19.96 to 136.19)	(10.93 to 19.18)	(−32.19 to 30.74)	(15.38 to 26.47)	(−24.11 to −43.94)	(9.46 to 16.55)	(−41.65 to 12.01)	(279.67 to 490.95)	(−46.17 to 4.98)
Pancreatic cancer	Both	3.64	150.43	2.49	13.62	3.77	73.51	1.85	−20.24	40.38	−23.24
		(2.50 to 4.70)	(85.57 to 226.38)	(1.71 to 3.21)	(−14.39 to 45.88)	(2.51 to 4.91)	(29.35 to 125.11)	(1.29 to 2.40)	(−39.19 to 2.21)	(28.21 to 52.61)	(−42.26 to 0.09)
	Female	3.44	119.44	2.17	−0.86	3.15	45.90	1.67	−28.05	35.83	−32.21
		(2.12 to 4.69)	(45.73 to 212.68)	(1.33 to 2.95)	(−33.49 to 40.79)	(1.91 to 4.30)	(−5.19 to 107.63)	(1.04 to 2.25)	(−50.12 to 2.74)	(22.37 to 48.65)	(−53.77 to −2.62)
	Male	3.82	184.70	2.89	29.01	4.47	101.20	2.11	−11.52	45.57	−13.79
		(2.21 to 5.35)	(90.45 to 310.65)	(1.69 to 4.00)	(−10.81 to 80.05)	(2.58 to 6.19)	(36.18 to 183.27)	(1.26 to 2.92)	(−37.77 to 23.48)	(26.80 to 63.81)	(−41.90 to 23.76)
Esophageal cancer	Both	8.34	159.46	5.64	24.18	4.53	27.34	5.72	18.36	137.23	11.42
		(6.65 to 10.17)	(87.37 to 246.22)	(4.52 to 6.84)	(−9.78 to 64.66)	(3.60 to 5.50)	(−8.43 to 69.75)	(4.59 to 6.91)	(−13.52 to 56.49)	(108.15 to 166.74)	(−20.12 to 49.29)
	Female	6.68	149.22	4.18	15.04	3.22	16.35	4.29	9.95	96.89	1.59
		(5.03 to 8.54)	(69.08 to 256.77)	(3.15 to 5.34)	(−21.86 to 63.55)	(2.43 to 4.14)	(−21.66 to 66.95)	(3.23 to 5.46)	(−25.20 to 56.00)	(72.71 to 125.18)	(−31.86 to 46.85)
	Male	9.93	167.08	7.29	31.28	5.92	35.58	7.37	24.77	179.36	18.52
		(7.46 to 12.77)	(77.71 to 279.62)	(5.55 to 9.24)	(−10.84 to 82.25)	(4.50 to 7.54)	(−8.77 to 89.96)	(5.64 to 9.30)	(−14.62 to 71.48)	(134.98 to 229.10)	(−20.73 to 66.90)

Abbreviations: CIR, crude incidence rate; ASIR, age-standardized incidence rate; ASDR, age-standardized death rate; ASPR, age-standardized prevalence rate; DALYs, disability-adjusted life years.

**Table 2:** APC model analysis of gastrointestinal cancer incidence for females and males in China.

	Esophageal Cancer		Stomach Cancer		Colon and Rectum Cancer		Liver Cancer		Gallbladder and Biliary Tract Cancer		Pancreatic Cancer	
	Male	Female	Male	Female	Male	Female	Male	Female	Male	Female	Male	Female
Longitudinal Age Effect												
Under 5	-	-	-	-	-	-	2.52	3.74	-	-	-	-
5–9	-	-	-	-	-	-	0.56	0.57	-	-	-	-
10–14	-	-	-	-	-	-	0.34	0.28	-	-	-	-
15–19	-	-	0.91	1.04	0.31	0.48	0.42	0.29	-	-	0.04	0.04
20–24	0.76	0.52	2.17	2.53	0.59	0.87	0.84	0.55	0.02	0.04	0.08	0.09
25–29	1.23	0.61	4.87	5.43	1.38	1.7	2.41	0.95	0.05	0.08	0.19	0.18
30–34	3.38	1.03	12.64	9.67	3.47	3.5	5.96	1.51	0.17	0.15	0.68	0.37
35–39	8.99	2.26	24.35	14.99	6.72	6.06	10.94	2.21	0.44	0.38	1.88	0.81
40–44	21.87	5.14	42.88	21.11	12.16	9.99	18.14	3.4	0.97	0.73	3.61	1.64
45–49	37.37	8.25	64.1	23.74	20.85	14.27	26.44	5.6	1.42	1.32	5.65	2.65
50–54	60.94	15.39	99.37	34.85	36.66	25.99	32.82	7.97	2.74	2.42	9.4	5.2
55–59	84.81	22.53	145.05	46.97	62.08	40.26	35.76	10.13	4.78	4.15	14.98	8.72
60–64	110.47	31.05	189.93	60.8	96.49	58.36	45.71	15.47	7.08	6.6	21.65	13.22
65–69	133.73	39.26	238.17	77.14	149.86	85.69	52.54	20.91	10.56	9.66	29.96	18.91
70–74	167.68	48.97	310.98	99.54	238.96	132.06	55.74	25.69	16.95	14.85	43.32	28.27
75–79	180.39	52.47	339.38	111.38	350.21	174.18	60.08	30.86	25.73	19.92	54.18	35.2
80–84	193.28	53.09	345.94	116.11	440.18	205.47	73.11	38.77	33.68	24.3	67.81	41.8
85–89	281.15	53.87	451.04	116.2	755.53	239.32	100.72	47.66	57.18	26.23	114.47	47.17
90–94	290.71	47.63	509.58	115.61	877.59	223.46	107.72	41.55	69.32	25.73	127.18	49.44
95+	152.16	39.93	275.02	111.74	558.06	223.43	83.57	27.54	38.94	24.17	80.92	41.89
Period Rate Ratio												
1992–1996	1.17	1.33	1.05	1.18	0.83	0.99	1.08	1.28	0.89	1.02	0.93	1.02
1997–2001	1.08	1.16	0.98	1.06	0.88	0.98	1.13	1.18	0.9	0.96	0.95	1.02
2002–2006 (base)	1	1	1	1	1	1	1	1	1	1	1	1
2007–2011	0.88	0.77	0.9	0.81	1.16	1.04	0.96	0.94	1.11	1	1.06	0.98
2012–2016	0.79	0.61	0.82	0.68	1.32	1.05	1.01	0.95	1.16	0.95	1.11	0.95
2017–2021	0.74	0.58	0.76	0.66	1.46	1.18	0.98	0.89	1.2	1	1.16	1.03
Cohort Rate Ratio												
1897	0.98	3.23	1.1	2.48	0.34	0.88	0.38	0.69	0.48	0.95	0.42	0.82
1902	1.29	2.85	1.33	2.26	0.44	0.8	0.67	1.04	0.64	0.92	0.55	0.77
1907	1.17	2.55	1.24	2.12	0.4	0.69	0.76	1	0.65	0.93	0.55	0.76
1912	1.21	2.28	1.28	1.96	0.42	0.65	0.7	0.75	0.71	0.92	0.62	0.77
1917	1.32	2.27	1.32	1.91	0.47	0.67	0.88	0.89	0.75	0.94	0.68	0.81
1922	1.32	2.13	1.31	1.82	0.52	0.72	1.05	0.99	0.77	0.98	0.74	0.88
1927	1.27	1.93	1.28	1.7	0.6	0.79	1.1	1.06	0.82	1.01	0.81	0.94
1932	1.19	1.66	1.21	1.52	0.69	0.86	1.05	1.06	0.87	1.02	0.86	0.98
1937	1.11	1.42	1.14	1.33	0.78	0.9	0.99	1.01	0.9	1.02	0.91	0.99
1942	1.06	1.22	1.06	1.14	0.86	0.94	0.94	0.96	0.93	0.99	0.94	0.99
1947	1	1	1	1	1	1	1	1	1	1	1	1
1952 (base)	0.82	0.7	0.89	0.82	1.12	1.04	1.01	0.98	1.04	0.97	1.02	0.97
1957	0.73	0.54	0.78	0.68	1.25	1.06	0.93	0.85	1.11	0.95	1.05	0.95
1962	0.63	0.45	0.67	0.56	1.37	1.04	0.94	0.8	1.12	0.89	1.03	0.88
1967	0.48	0.4	0.64	0.5	1.61	1.07	0.96	0.77	1.22	0.88	1.09	0.85
1972	0.42	0.27	0.55	0.42	1.74	1.06	0.9	0.66	1.34	0.88	1.07	0.81
1977	0.35	0.22	0.53	0.38	2.08	1.09	0.84	0.55	1.63	0.91	1.17	0.79
1982	0.32	0.21	0.53	0.36	2.46	1.12	0.82	0.48	1.93	0.92	1.3	0.77
1987	0.32	0.19	0.57	0.35	2.89	1.16	0.82	0.45	2.25	0.93	1.45	0.77
1992	0.29	0.17	0.56	0.33	3.09	1.2	0.73	0.39	2.49	0.97	1.46	0.79
1997	0.31	0.14	0.52	0.29	3.19	1.16	0.63	0.34	2.83	0.98	1.3	0.9
2002	-	-	0.4	0.23	2.97	1.03	0.5	0.25	-	-	1.24	0.58
2007	-	-	-	-	-	-	0.38	0.2	-	-	-	-
2012	-	-	-	-	-	-	0.3	0.16	-	-	-	-
2017	-	-	-	-	-	-	0.26	0.13	-	-	-	-
Net Drift	−1.8949	−3.5547	−1.3031	−2.5053	2.4166	0.6315	−0.4953	−1.4078	1.3608	−0.0834	0.9427	−0.1082
*p*-Value	0	0	0.019	0	0	0	0.019	0	0	0.253	0	0.293

## Data Availability

The dataset used for this manuscript is a publicly available dataset available in the Global Burden of Disease Study 2021 (GBD 2021) Data Resources. The dataset is downloadable for research purposes at the following link: https://ghdx.healthdata.org/gbd-2021 (accessed on 16 May 2024).

## Supplementary Materials

The following supporting information can be downloaded at https://www.mdpi.com/article/10.3390/healthcare13101096/s1: Figure S1: Predicted age-specific incidence rate of gastrointestinal cancers in 1990–2050. Figure S1a–l shows the prediction results of cancer burden for esophageal cancer, stomach cancer, colon and rectum cancer, liver cancer, gallbladder and biliary tract cancer, pancreatic cancer in males and females.
