# Supplementary material for: Age-Period-Cohort Analysis on the Burden of Gastrointestinal Cancers in China: Trends, Risk Factors, and Predictions
Source: Healthcare (Basel). 2025 May 8;13(10):1096. doi: 10.3390/healthcare13101096 (PMC12110969; doi:10.3390/healthcare13101096)

Figure S1. Predicted age-specific incidence rate of gastrointestinal cancers in 1990-2050

Figure S1a. Predicted age-specific incidence rate of esophageal cancer for male in 1990-2050

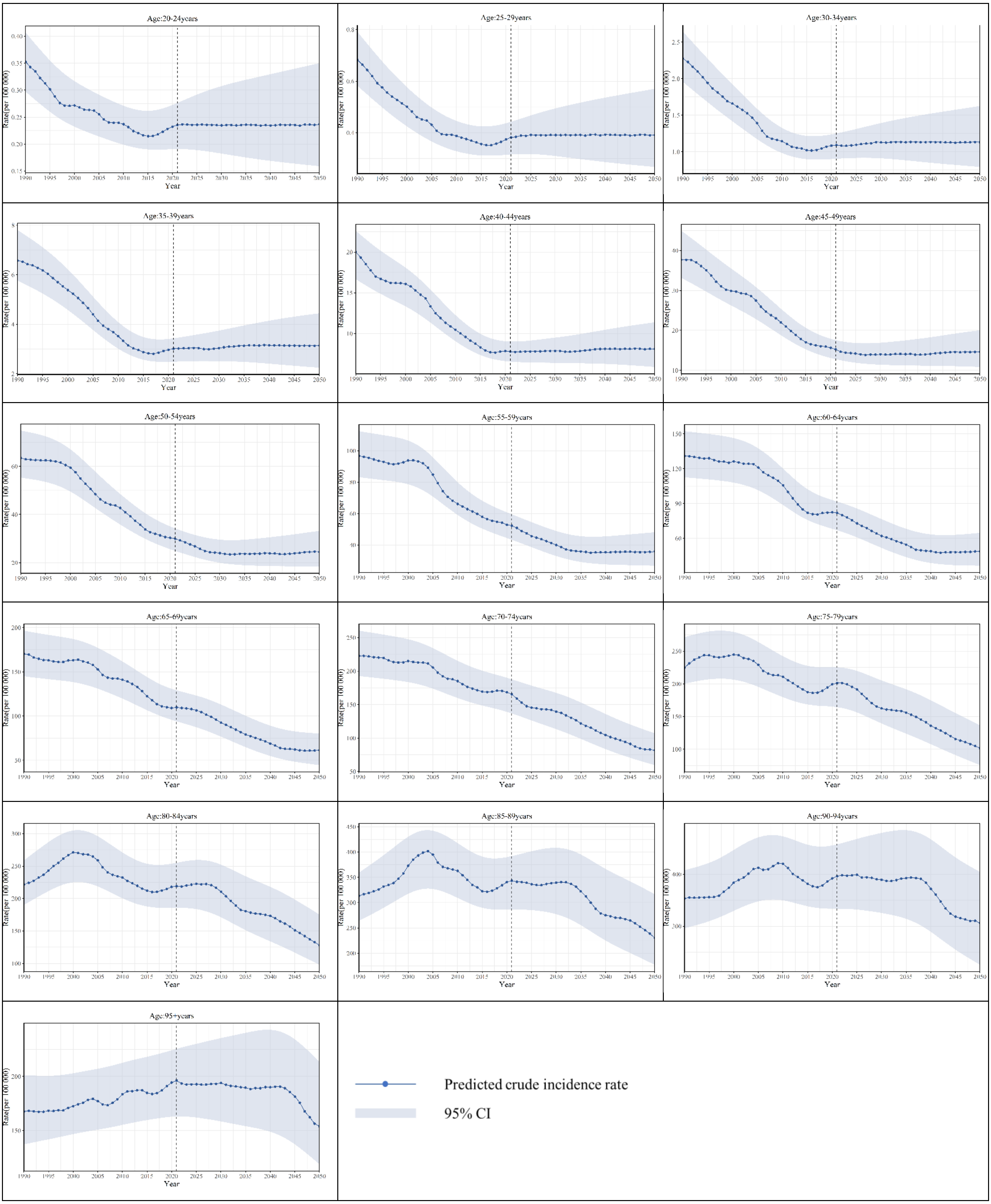

Figure S1b. Predicted age-specific incidence rate of esophageal cancer for female in 1990-2050

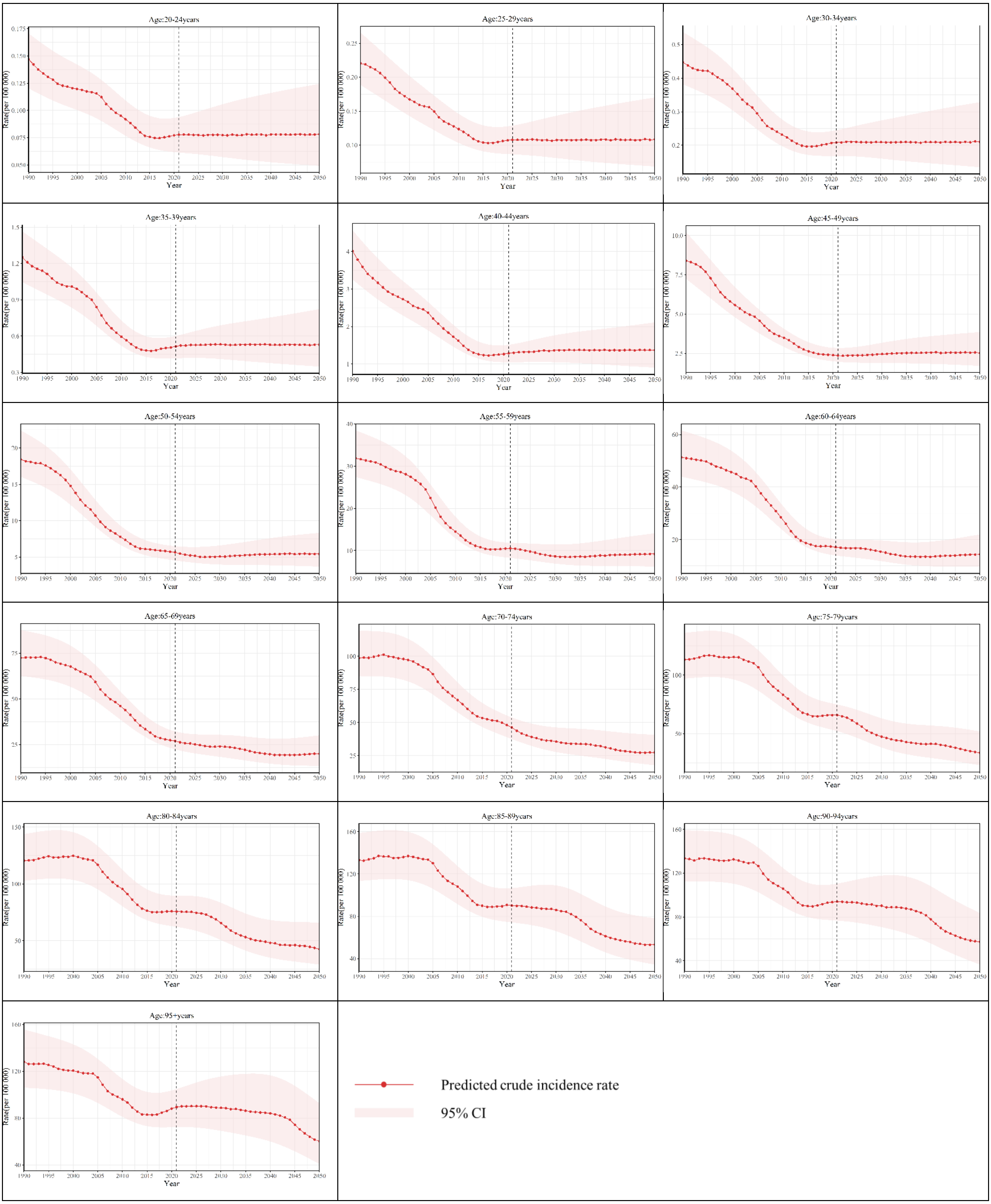

Figure S1c. Predicted age-specific incidence rate of stomach cancer for male in 1990-2050

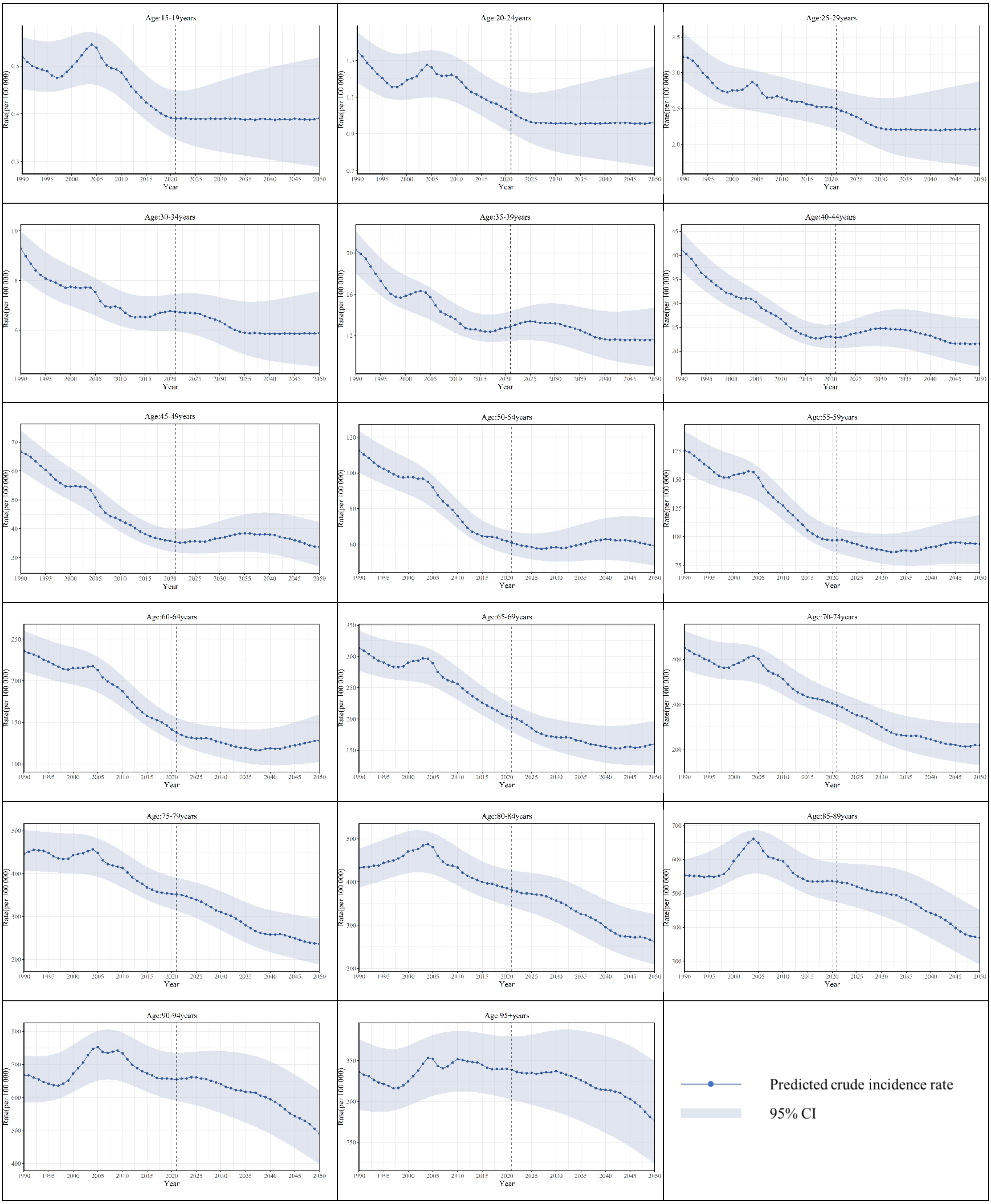

Figure S1d. Predicted age-specific incidence rate of stomach cancer for female in 1990-2050

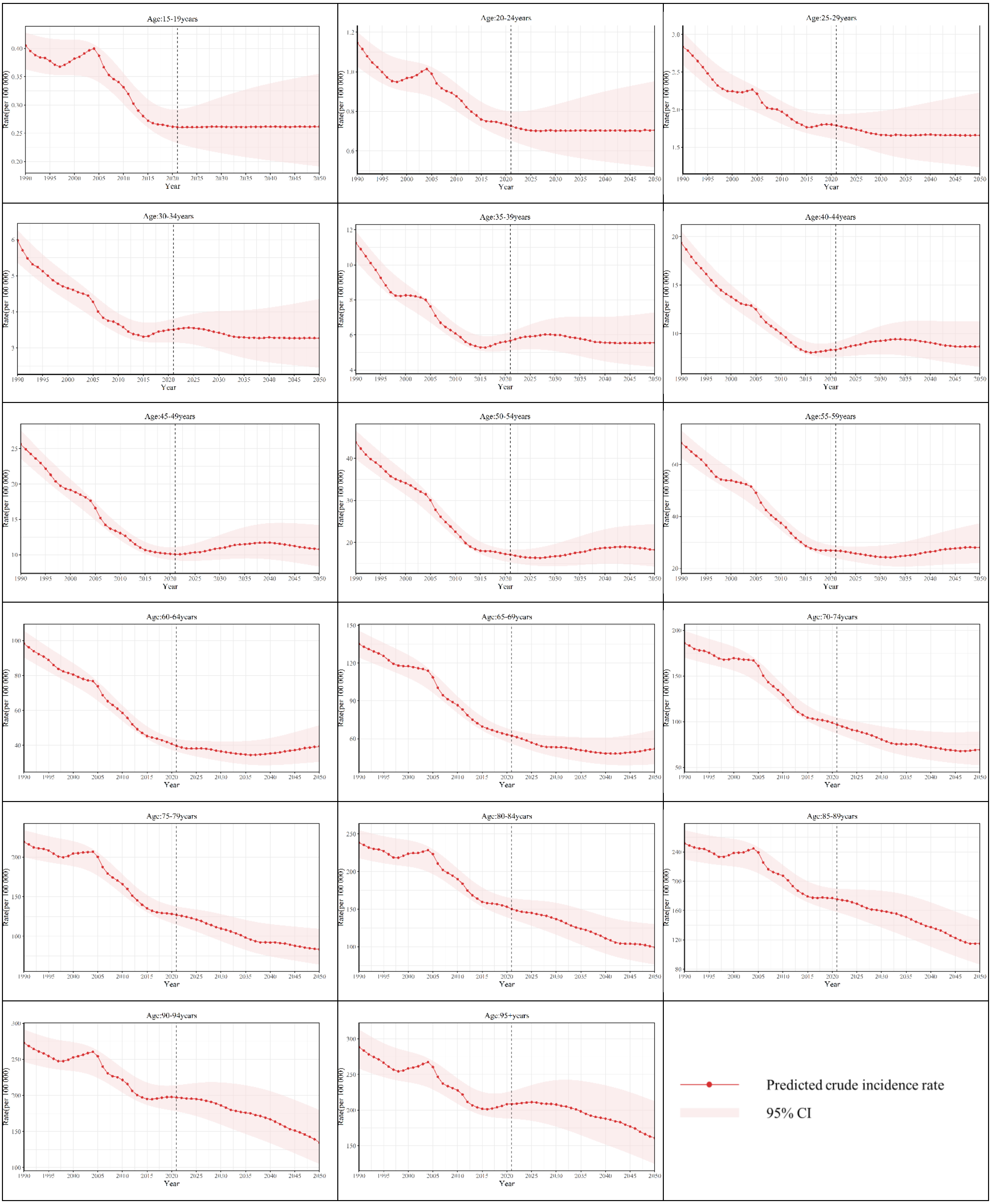

Figure S1e. Predicted age-specific incidence rate of colon and rectum cancer for male in 1990-2050

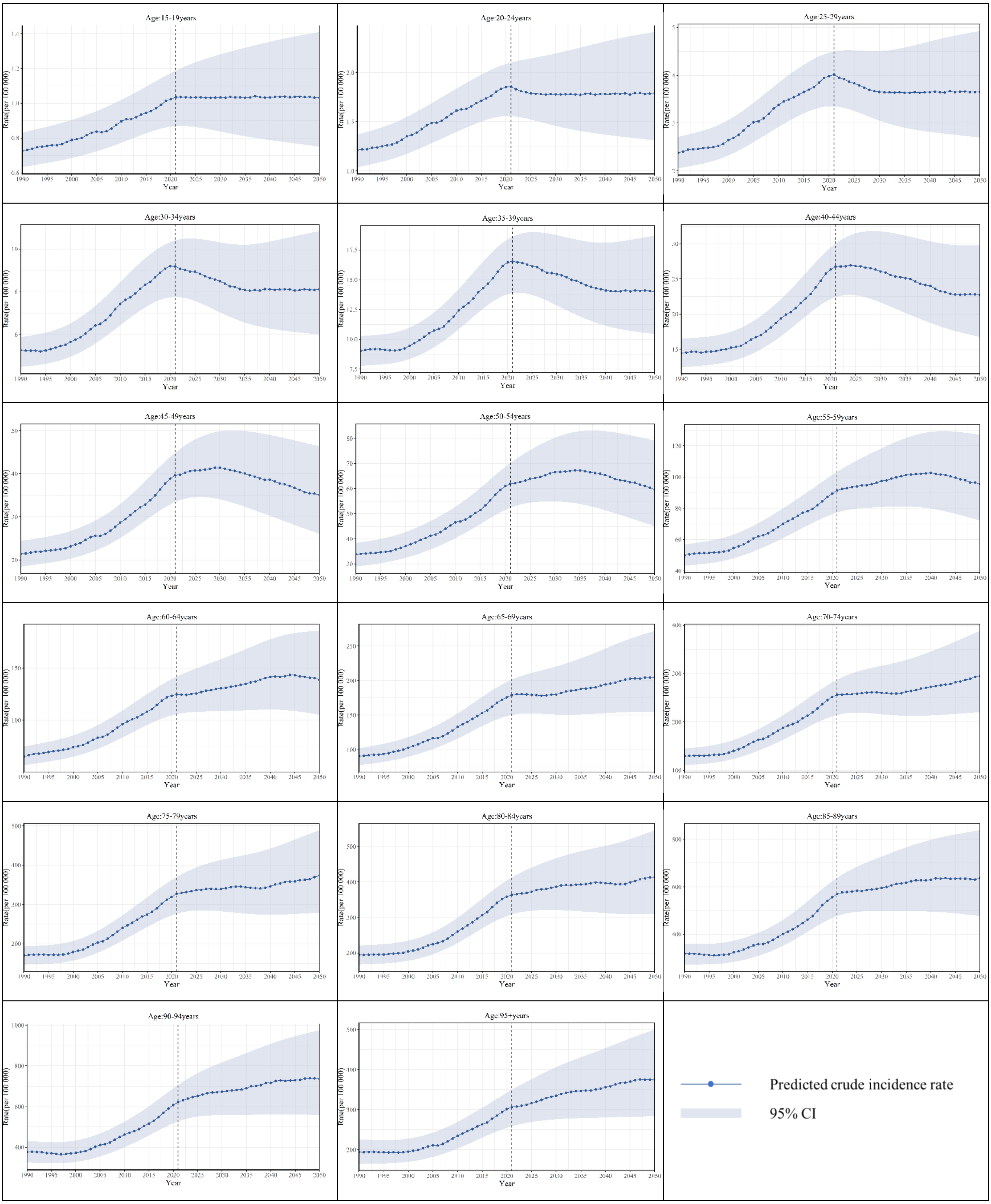

Figure S1f. Predicted age-specific incidence rate of colon and rectum cancer for female in 1990-2050

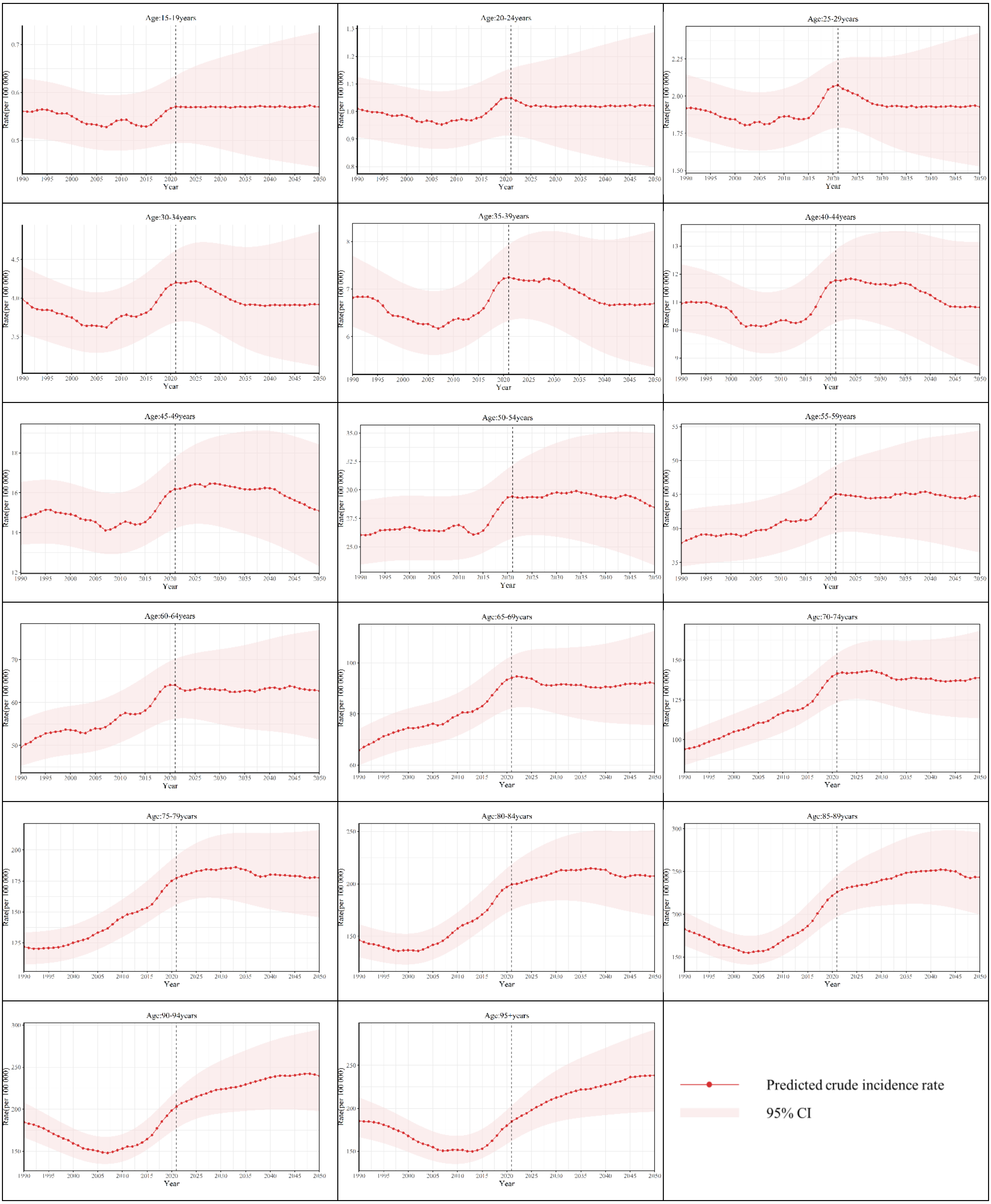

Figure S1g. Predicted age-specific incidence rate of liver cancer for male in 1990-2050

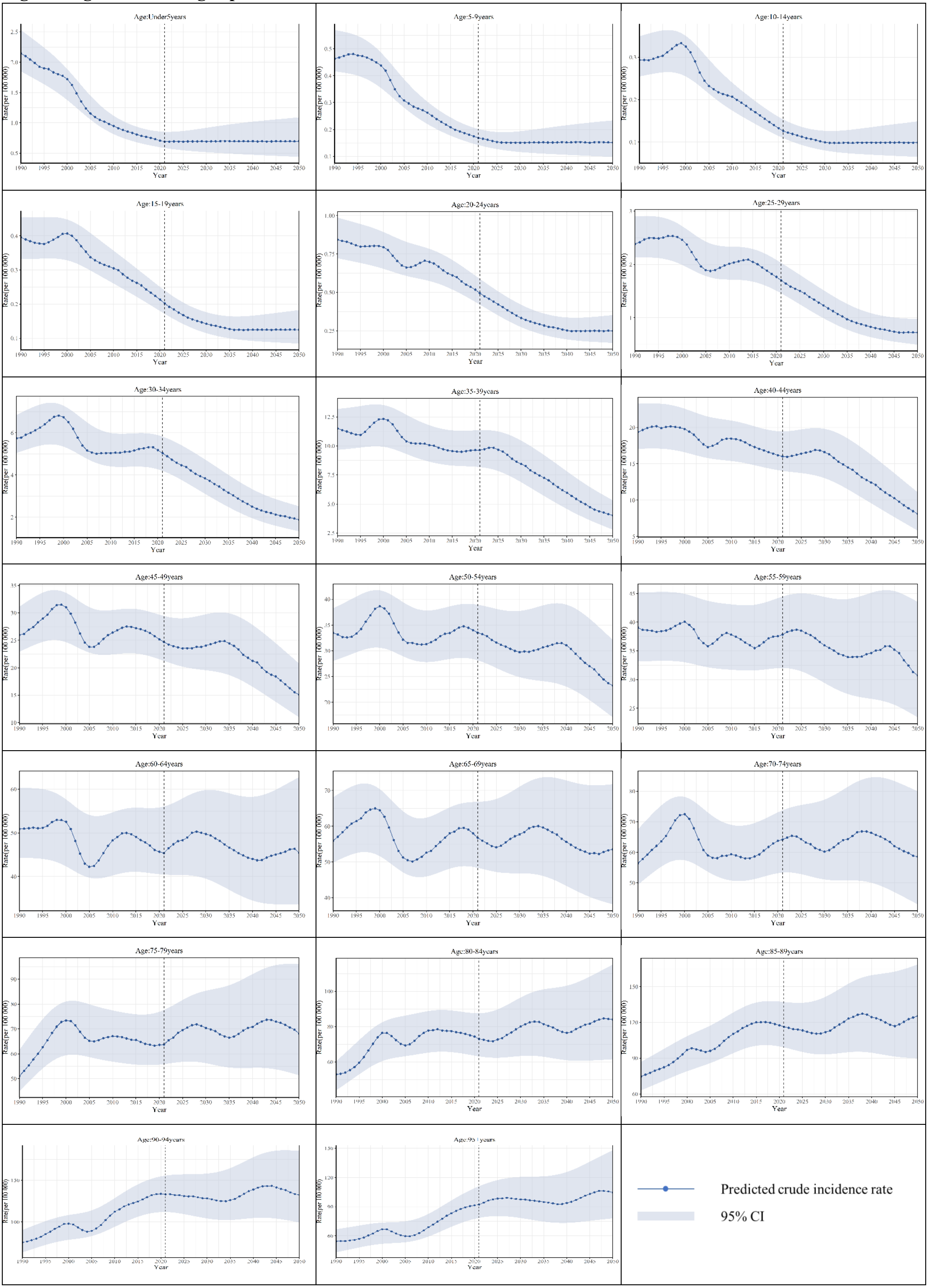

Figure S1h. Predicted age-specific incidence rate of liver cancer for female in 1990-2050

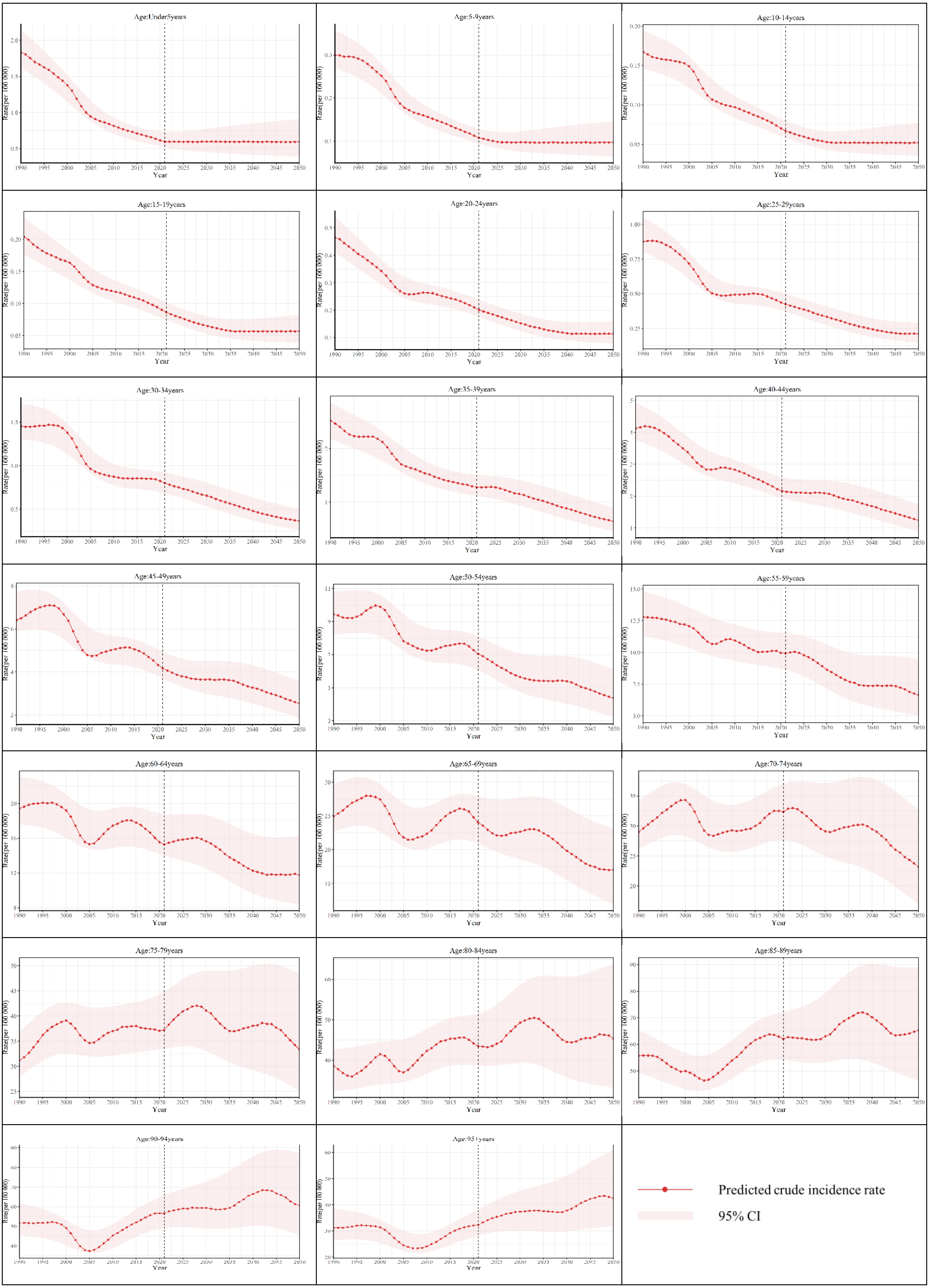

Figure S1i. Predicted age-specific incidence rate of gallbladder and biliary tract cancer for male in 1990-2050

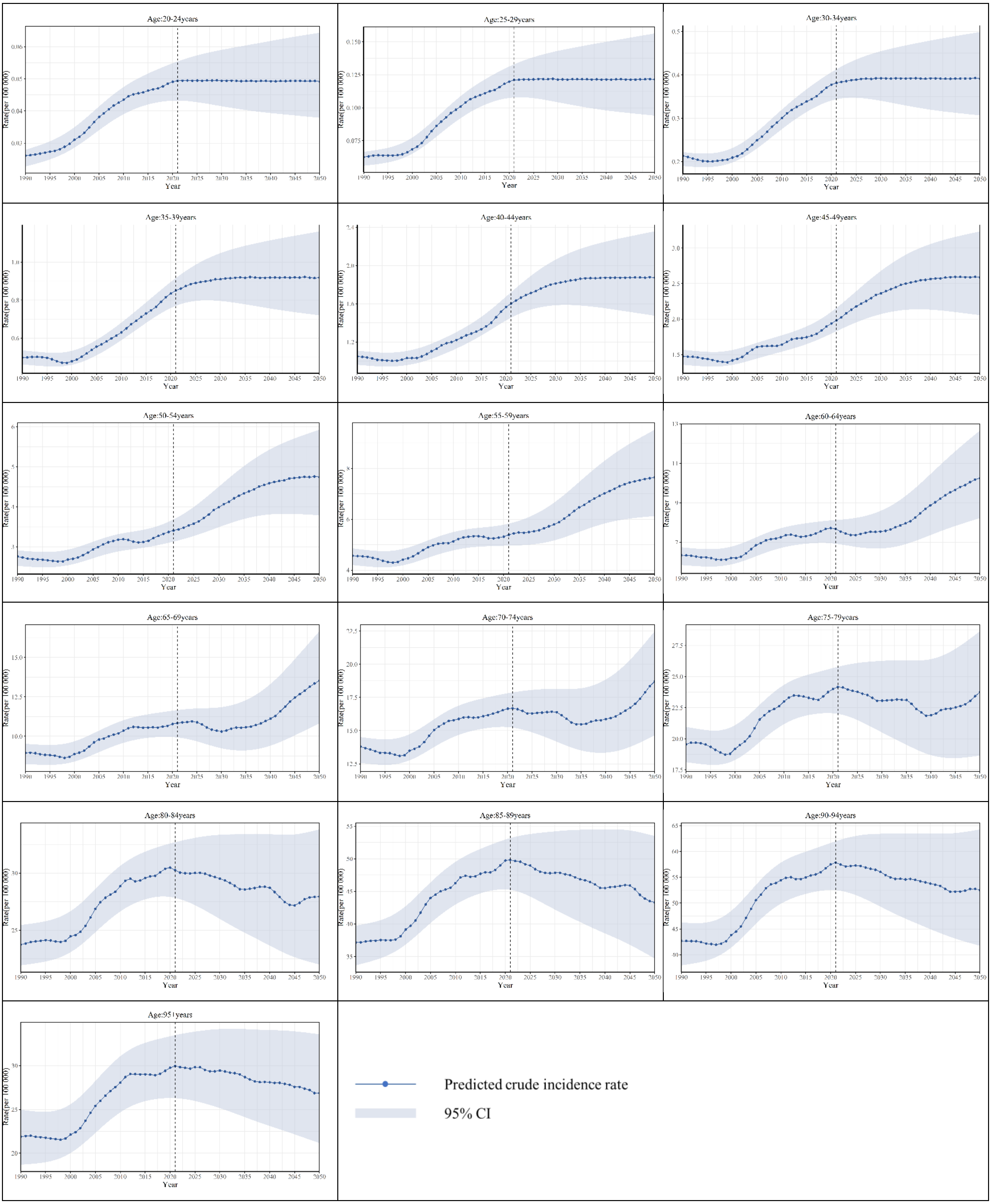

Figure S1j. Predicted age-specific incidence rate of gallbladder and biliary tract cancer for female in 1990-2050

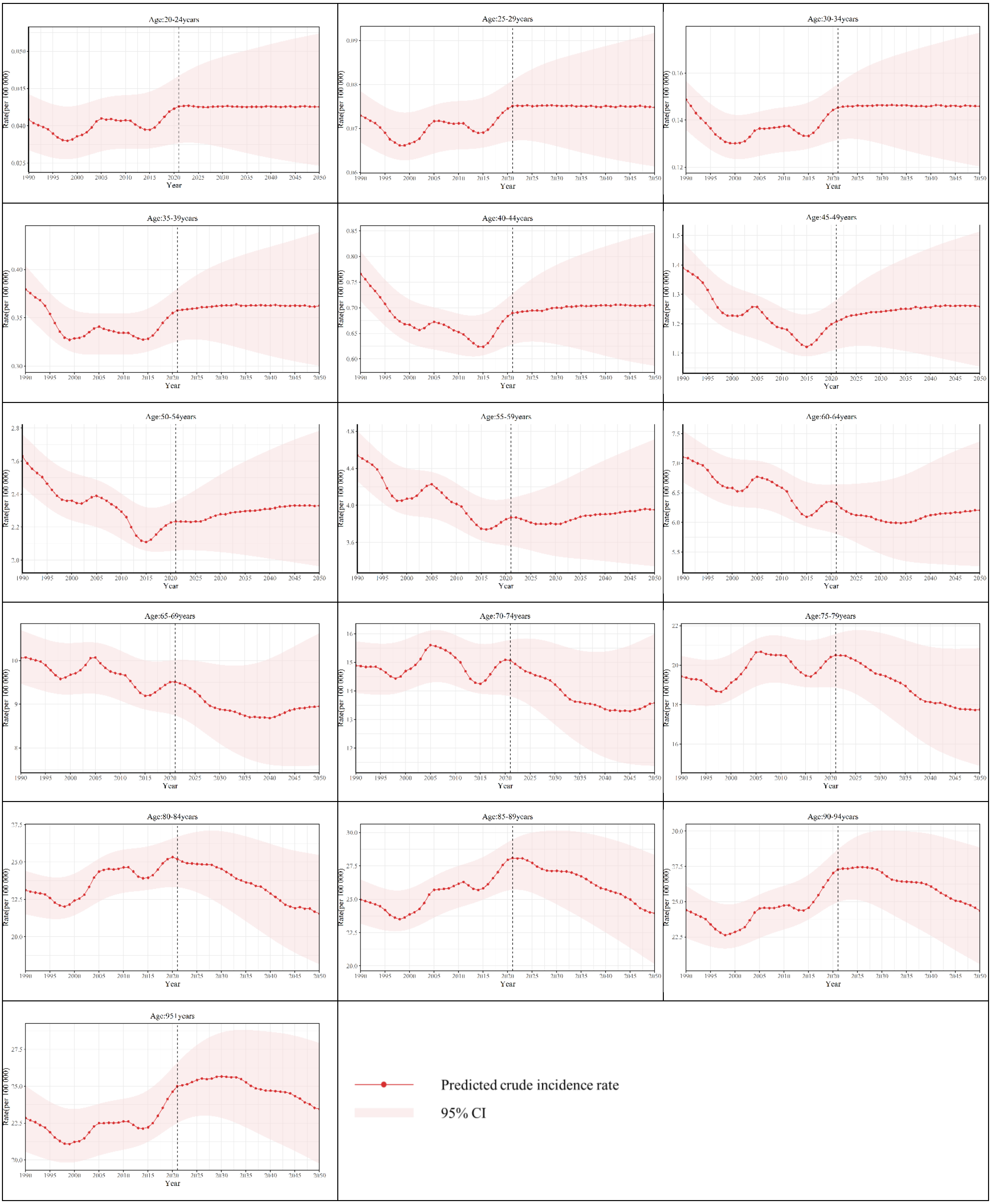

Figure S1k. Predicted age-specific incidence rate of pancreatic cancer for male in 1990-2050

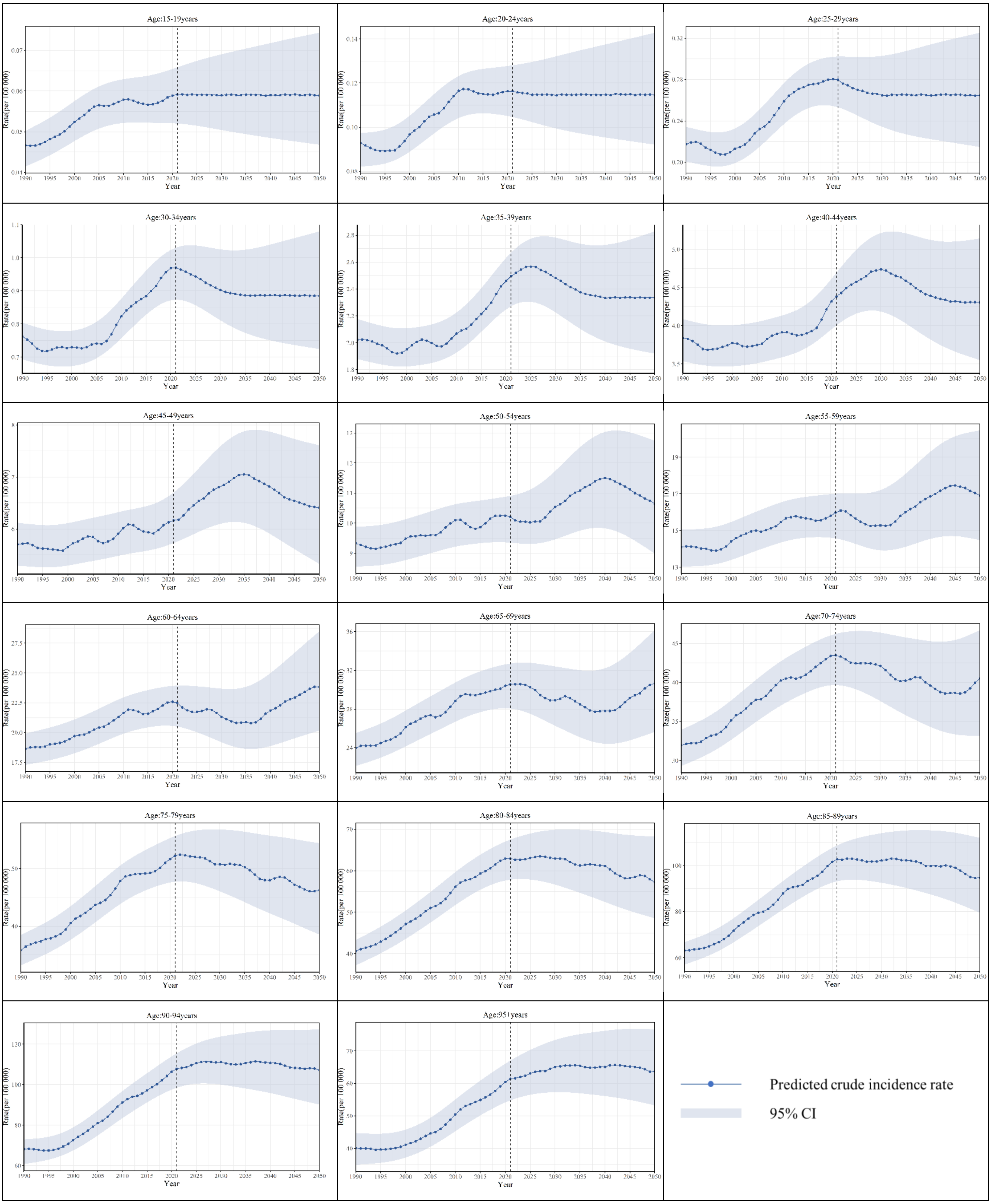

Figure S1l. Predicted age-specific incidence rate of pancreatic cancer for female in 1990-2050

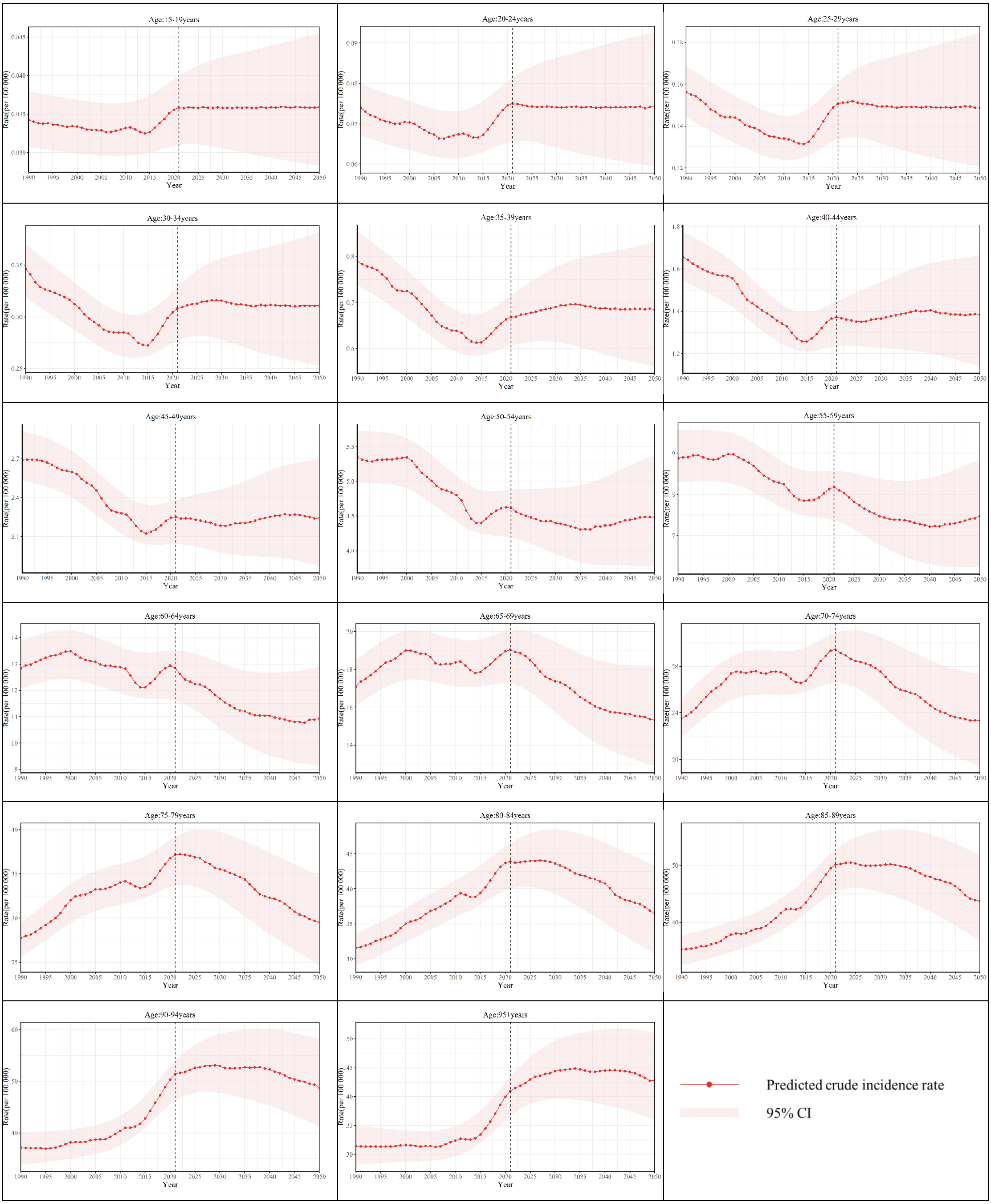

Supplement: Supplementary file 1 [file healthcare-13-01096-s001.zip › healthcare-3510785-supplementary.pdf]
